# Hemadsorption to Contain Postoperative Cell-Free Hemoglobin and Haptoglobin Preservation for Extended Cardiopulmonary Bypass Time in Cardiac Surgery for Acute Kidney Injuries Prevention

**DOI:** 10.21470/1678-9741-2023-0272

**Published:** 2024-05-13

**Authors:** Ignazio Condello, Juan Blanco Morvillo, Flavio Fiore, Valentina Teora, Giuseppe Nasso, Giuseppe Speziale

**Affiliations:** 1 Department of Cardiac Surgery, Anthea Hospital, GVM Care & Research, Bari, Italy; 2 Department of Cardiac Surgery, Biomedic Research Institute of Murcia (IMIB), Virgen de la Arrixaca University Hospital, Murcia, Spain

**Keywords:** Hemadsorption, Cardiopulmonary Bypass, Haptoglobin, Hemolysis, Acute Kidney Injury

## Abstract

**Introduction:**

Prevention of acute kidney injury during cardiopulmonary bypass (CPB) is still a challenge and has been the object of numerous studies. The incidence of acute kidney injury in the context of CPB is related to a multifactorial etiology. The role of hemadsorption in relation to cell-free hemoglobin and haptoglobin preservation is not well defined in the literature on CPB during cardiac surgery procedures.

**Methods:**

This is a single-center pilot randomized report including 20 patients undergoing elective CPB procedures with an expected time > 120 minutes for each extracorporeal procedure. Patients were randomly allocated to either standard of care (n=10) or Jafron HA380 (n=10) during CPB. The primary outcome measured was the incidence of postoperative acute kidney injuries.

**Results:**

The Jafron study group vs. control group reported postoperative values for cell-free hemoglobin at 10 minutes after CPB (mg/L) (11.6 ± 0.6 vs. 29.9 ± 0.3) (P-value 0.021), haptoglobin 10 minutes after CPB (mg/dl) (129.16 ± 1.22 vs. 59.17 ± 1.49) (P-value 0.017), creatinine peak after CPB (mg/dL) (0.92 ± 0.17 vs. 1.32 ± 0.9) (P-value 0.030), and acute kidney injury after 48 hours (number of patients) (one vs. four) (P-value 0.027).

**Conclusion:**

This pilot study suggested that the use of Hemoperfusion Cartridge HA380 Jafron for extended CPB time for complex cardiac surgery procedures was safe and effective and is associated with a better postoperative preservation of haptoglobin with a reduction of cell-free hemoglobin values and less incidence of acute kidney injury, though larger studies are warranted to confirm our result.

## INTRODUCTION

**Table:** 

Abbreviations, Acronyms & Symbols				
AKI	= Acute kidney injury		EuroSCORE	= European System for Cardiac Operative Risk Evaluation
AKIN	= Acute Kidney Injury Network		HA	= Hemadsorption
CFH	= Cell-free hemoglobin		Hb	= Hemoglobin
CI	= Cardiac index		HTC	= Hematocrit
COVID-19	= Coronavirus disease 2019		MAP	= Mean arterial pressure
CPB	= Cardiopulmonary bypass		SEM	= Standard error of mean
DO_2_	= Oxygen delivery		VAVD	= Vacuum-assisted venous drainage
DO_2i_	= Indexed oxygen delivery		VCO_2_	= Carbon dioxide production

Cardiopulmonary bypass (CPB) is an indispensable technique used in open-heart surgery that ensures perfusion of vital organs and metabolism support. While essential, CPB can induce the massive release of inflammatory cytokines, causing overwhelmed systemic inflammation reactions that may increase perioperative mortality^[1]^. The role of hemadsorption (HA) and of the relative medical devices for cytokine storm attenuation has been widely described in the literature, although there are conflicting opinions on the use of them during treatment of septic shock and on their application in extracorporeal techniques in terms of survival and mortality with respect to cost benefit. Extracorporeal blood purification can be achieved by different mass separation processes^[2]^ - diffusion, as in standard hemodialysis, convection, as in hemofiltration, or their combination, as in hemodiafiltration. While these techniques are based on membrane separation, a third mechanism, solute adsorption, is based on mass separation by a solid agent (sorbent)^[3]^, which was used especially in Coronavirus disease 2019 (COVID-19) pandemic, as the systemic inflammatory reaction caused by the virus was one of the main causes of death^[4]^. Within this scenario, HA gained prominence due to the possibility of reducing circulating cytokines and, possibly, reducing the inflammatory response^[5]^. These attempts were based on the benefits produced in cases of poisoning or even autoimmune diseases^[6]^. Two sorbent technologies have emerged: the CytoSorb® cartridges and the Jafron HA cartridges series. These sorbents have been used as rescue therapy in sepsis or as adjuvant therapy in sepsis, and experience has accumulated in terms of technique and safety. Increased plasma concentrations of circulating cell-free hemoglobin (CFH) during CPB are supposed to contribute to the multifactorial etiology of acute kidney injury (AKI). Among patients with increased CFH concentrations, higher plasma haptoglobin concentrations might protect from CFH-associated AKI^[7]^. The role of HA in relation to CFH and haptoglobin preservation is not well defined in the literature on CPB during cardiac surgery procedures. In this context, we introduce a preliminary report aiming to evaluate the potential of Hemoperfusion Cartridge HA380 Jafron to decrease perioperative CFH values and haptoglobin preservation for extended CPB time in cardiac surgery and its correlation with postoperative kidney injuries.

## METHODS

### Population and Study Design

**Fig. 1:**
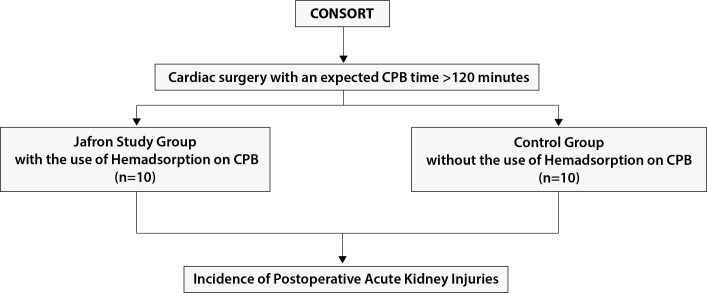
CONSORT diagram. CPB=cardiopulmonary bypass.

Between March 2023 and May 2023, 20 patients aged > 18 years with a mean European System for Cardiac Operative Risk Evaluation (EuroSCORE) II of 3.9-4.1% and left ventricular ejection fraction > 40% underwent cardiac surgery procedures at our institution with an expected CPB time > 120 minutes. The local Ethics Committee approved this study (protocol 0023296), ClinicalTrials.gov Identifier is NCT05349669, and all patients provided written informed consent to data treatment. Patients with chronic renal failure, type 1 or 2 diabetes mellitus, septic shock or endocarditis, and with hemoglobin values < 8 g/dl before the procedure were excluded. A perspective data collection was performed on 20 randomized consecutive patients who underwent cardiac surgery procedures: 10 were allocated with Hemoperfusion Cartridge HA380 Jafron use during CPB (Jafron study group), and 10 were allocated without the use of HA on CPB (control group) (Figure 1). The primary parameters collected were preoperative patient characteristics, perioperative parameters (CPB time, cross-clamping time, mean arterial pressure [MAP], indexed oxygen delivery [DO_2i_], surgical procedures, and cardiac index), and postoperative haptoglobin, CFH, and creatinine values^[7,8]^. The primary outcome measured was the incidence of postoperative AKI, which we defined as the peak postoperative serum creatinine value and the presence of AKI according to the AKI Network (AKIN) criteria^[9]^. Briefly, a patient was assigned to the AKI stage 1 group based on an increase in peak postoperative serum creatinine ≥ 150% to 200% from the baseline value and to the AKI stage 2 group based on an increase in peak postoperative serum creatinine > 200% to 300% from the baseline value. Patients assigned to AKI stage 3 (peak postoperative serum creatinine value more than three times the baseline value) were identified but included in the AKI stage 2 group because of the predictable low rate of events. According to the AKIN criteria, the assignment of patients to the different AKI stages was based on creatinine changes only, and urine output was not considered. Creatinine changes were recorded within the first 48 hours after the operation.

### Cardiopulmonary Bypass Setting

Only the open system (Horizon AF PLUS venous reservoir and oxygenator, Eurosets, SRL, Medolla, Italy) was used for CPB. All patients were treated with mild hypothermic CPB (34-36°C); a volume of 1250 mL crystalloid Ringer acetate solution was used for priming. The surgical procedures selected for this study do not justify the use of moderate hypothermia by falling < 34°C. For this reason, in the event of an initial increase in anaerobic metabolism, the first compensation approach was not to lower the temperature; however, possibly liquids or red blood cells were integrated. The hardware consisted of a Stöckert S5 heart-lung machine and a Stöckert 3T heater-cooler system (LivaNova), and the same cannulae were employed in both groups. The venous drainage line (3/8 inch) and the arterial delivery line (3/8) were each 180 cm long, the lines to the pump (3/8 and 1/2) were each 80 cm long, and the cardioplegia line (1/16) was 190 cm long. The aspiration lines were 1/4. This circuit uses a serial pump with vacuum-assisted venous drainage (VAVD). Roller pumps were used because aspiration has a management nadir < 800 mL/min to > 2 L/min. A negative pressure of -40 mmHg VAVD was applied to the reservoir. The intracavitary aspirator managed with a roller pump was channeled into a venous reservoir, and the extracavitary aspirator was managed with a roller pump^[3]^. The landing monitoring system (Eurosets, SRL, Medolla, Italy) was used for oxygen delivery (DO_2_) management during CPB. Metabolic parameters were monitored with a DO_2i_ system; the nadir was > 280 mL/min/m^2^. The security system used a level alarm, and a bubble probe was used to detect microbubbles leaving the venous reservoir. Anticoagulant therapy consisted of heparin sodium before CPB at 300 IU/kg to give an activated clotting time > 480 s. Cardioplegia was performed in an antegrade manner with normothermic blood in a 190 cm closed circuit with a bubble-trap filter by a serial micrometric pump, with St. Thomas solution with procaine, and repeated every 30 minutes^[8]^. In the Jafron study group, the hemoperfusion cartridge HA380 Jafron was prepared before the CPB institution following the protocol and instructions of the Handbook l Device.

### Anaesthetics and Surgical Procedures

Patients were monitored with five-lead electrocardiography, a left radial artery catheter, capnography, pulse oximeter, and rectal/urine bladder temperature sensors. Transesophageal echocardiography was performed in all patients. Anaesthesia was induced with intravenous sufentanil (0.5-1 µg/kg) and midazolam (0.08-0.2 mg/kg), and tracheal intubation was facilitated with intravenous rocuronium (0.6-1 mg/kg)^[2]^. Anaesthesia was maintained with propofol (2-5 mg/kg) and sufentanil (0.5-2.0 µg/kg), and the depth of anaesthesia was monitored using bispectral index values (BIS XP, Aspect Medical System, Newton, Massachusetts, United States of America). The dosage of propofol was titrated to maintain bispectral index values between 40 and 45. All operations were performed with median sternotomy.

### Statistical Analysis

Continuous data were expressed as mean ± standard deviation or a median with the interquartile range and categorical data as percentages. Cumulative survival was evaluated with the Kaplan-Meier method. All reported *P*-values were two-sided, and *P*-values < 0.05 were considered to indicate statistical significance. All statistical analyses were performed with IBM Corp. Released 2013, IBM SPSS Statistics for Windows, version 22.0, Armonk, NY: IBM Corp.

## RESULTS

Patients’ characteristics and perioperative and postoperative results are described in Table 1. No deaths were recorded in the 20 patients in the postoperative period. The Jafron study group *vs.* control group reported postoperative values for CFH at 10 minutes after CPB (mg/L) = (11.6 ± 0.6 *vs.* 29.9 ± 0.3) (*P*-value 0.021), haptoglobin 10 minutes after CPB (mg/dl) = (129.16 ± 1.22 *vs.* 59.17 ± 1.49) (*P*-value 0.017), creatinine peak after CPB (mg/dL) = (0.92 ± 0.17 *vs.* 1.32 ± 0.9) (*P*-value 0.030), and AKI I after 48 hours (number of patients) = (one *vs.* four) (*P*-value 0.027) (Table 1).

**Table 1:** Preoperative, intraoperative, and postoperative data.

Procedures (n=20)	Jafron study group (*n*=10)	Control group (*n*=10)	*P*-value
Preoperative data			
Age (years)	61 ± 7	65 ± 5	0.89
Body surface area (m^2^)	1.83	1.82	0.94
EuroSCORE II	1.5	1.7	0.88
Pre-CPB hematocrit (%) (mean ± SEM)	34.6 ± 1.3	34.8 ± 2.1	0.99
Hb (g/dL)	12.3 ± 1.1	12.3 ± 1.2	1
Serum creatinine (g/dL)	0.83 ± 0.5	0.85 ± 0.7	0.96
Male sex	4	6	0.84
CFH (mg/L)	0.02	0.01	1
Haptoglobin (mg/dl)	164.16 ± 1.7	159.17 ± 1.5	0.78
Procedures			
Ascending aorta and aortic valve replacement	3	4	
Mitral valve repair and aortic valve replacement	4	3	
Mitral valve repair, tricuspid valve repair, and aortic valve replacement	3	3	
Intraoperative data			
CPB time	128 ± 6	123 ± 5	0.092
Aortic cross-clamping time (min)	102 ± 7	106 ± 4	0.93
DO₂_i_ (mL/min/m^2^)	289 ± 19	284 ± 13	0.99
CI (L/min/m^2^)	2.6 ± 0.4	2.5 ± 0.2	0.99
Hct (%)	34 ± 2	32 ± 1	0.69
Hb (g/dL)	11.5 ± 0.5	11.3 ± 0.6	0.76
MAP (mmHg)	63 ± 7	62 ± 4	0.91
Postoperative data			
CFH at 10 min. after CPB (mg/L)	11.6 ± 0.6	29.9 ± 0.3	0.021
Haptoglobin 10 min. after CPB (mg/dl)	129.16 ± 1.22	59.17 ± 1.49	0.017
Creatinine peak after CPB (mg/dL)	0.92 ± 0.17	1.32 ± 0.9	0.030
AKI after 48 h (number of patients)	1	4	0.027

Values are presented as n (%) or mean ± standard deviation

AKI=acute kidney injury; CFH=cell-free hemoglobin; CI=cardiac index; CPB=cardiopulmonary bypass; DO_2i_=indexed oxygen delivery; EuroSCORE=European System for Cardiac Operative Risk Evaluation; Hb=hemoglobin; HTC=hematocrit; MAP=mean arterial pressure; SEM=standard error of mean

## DISCUSSION

The modulation of the “cytokine storm” in COVID-19 pandemic seems to determine endothelial protection, which can translate into a reduction of the “capillary leak syndrome”, and, consequently, a better control of edema formation and pulmonary infiltrates^[10]^. However, in all these situations, the literature lacks more robust experiences to produce more consistent results. Following this same reasoning, speculations began to emerge on the advantages of extending the principles of HA to cardiovascular surgery with the use of CPB.

The prevention of AKI during CPB is still a challenge today and has been the object of numerous studies. The incidence of AKI in the context of CPB is related to a multifactorial etiology, which was often addressed in previous studies and articles by analyzing the single indexed variables of DO₂, carbon dioxide production (VCO₂), MAP, and micro-embolic activity^[8,11]^. De Somer et al.^[9]^ reported a nadir DO₂ level < 262 mL/minute/m^2^ and a nadir DO₂/VCO₂ ratio < 5.3 independently associated with AKI within a model including EuroSCORE and CPB duration. Increased plasma concentrations of circulating CFH are supposed to contribute to the multifactorial etiology of AKI. The importance of protective mechanisms against the adverse effects of intravascular hemolysis in organisms with a blood circulation is highlighted by the evolutionary early appearance and conservation of haptoglobin. CFH may also originate from direct mechanical injury of red cells in the vasculature. In addition, extracorporeal therapeutic measures such as CPB an extracorporeal membrane oxygenation can cause mechanical hemolysis. The injurious potential of intravascular CFH manifests most prominently in the kidney. In cardiac surgery patients, increased plasma concentrations of CFH after prolonged CPB are associated with AKI^[12]^.We presented a pilot study on Hemoperfusion Cartridge HA380 Jafron for extended CPB time with the aim of demonstrating that the hemolytic aspect in the control group for prolonged CPB (> 120 minutes) is able to influence and increase the incidence of AKI^[6]^; in this context, the use of HA in the study group was crucial for the reduction of circulating CFH values and the maintenance of haptoglobin values, especially for patients undergoing extensive cardiac surgery procedures involving the use of multiple roller aspirators^[11,13]^. M. Bernardi et al.^[7]^ demonstrated that HA hasn’t increased hemolysis in patients treated with cartridge. T. Gleason et al.^[1]^, in Refresh I pilot study, concluded that treatment with HA resulted in significant reductions in CFH during valve replacement surgery^[4]^. Our study with Jafron cartridge aligns perfectly with the experience reported by previous authors Bernardi and Gleason with the CytoSorb® with cartridge.

### Limitations

The main limitation of this pilot study is that is a single-center study with a small population, however, it is a study that presents a homogeneity in the preoperative and periprocedural variables.

## CONCLUSION

This pilot study suggested that the use of Hemoperfusion Cartridge HA380 Jafron for extended CPB time for complex cardiac surgery procedures is associated with a better postoperative preservation of haptoglobin with a reduction of CFH values and less incidence of AKI, though larger studies are warranted to confirm our result.

## References

[1] Gleason TG, Argenziano M, Bavaria JE, Kane LC, Coselli JS, Engelman RM (2019). Hemoadsorption to reduce plasma-free hemoglobin during cardiac
surgery: results of REFRESH I pilot study. Semin Thorac Cardiovasc Surg.

[2] Nierhaus A, Morales J, Wendt D, Scheier J, Gutzler D, Jarczak D (2022). Comparison of the cytoSorb® 300 mL and jafron HA380
hemoadsorption devices: an in vitro study. Minim Invasive Ther Allied Technol.

[3] Ronco C, Bellomo R. (2022). Hemoperfusion: technical aspects and state of the
art. Crit Care.

[4] Shi Y, Wang Y, Shao C, Huang J, Gan J, Huang X (2020). COVID-19 infection: the perspectives on immune
responses. Cell Death Differ.

[5] Chen N, Zhou M, Dong X, Qu J, Gong F, Han Y (2020). Epidemiological and clinical characteristics of 99 cases of 2019
novel coronavirus pneumonia in Wuhan, China: a descriptive
study. Lancet.

[6] Wang D, Hu B, Hu C, Zhu F, Liu X, Zhang J (2020). Clinical characteristics of 138 hospitalized patients with 2019
novel coronavirus-infected pneumonia in Wuhan, China. JAMA.

[7] Bernardi MH, Rinoesl H, Ristl R, Weber U, Wiedemann D, Hiesmayr MJ. (2019). Hemoadsorption does not have influence on hemolysis during
cardiopulmonary bypass. ASAIO J.

[8] Condello I, Santarpino G, Nasso G, Moscarelli M, Fiore F, Speziale G. (2020). Associations between oxygen delivery and cardiac index with
hyperlactatemia during cardiopulmonary bypass. JTCVS Tech.

[9] de Somer F, Mulholland JW, Bryan MR, Aloisio T, Van Nooten GJ, Ranucci M. (2011). O2 delivery and CO2 production during cardiopulmonary bypass as
determinants of acute kidney injury: time for a goal-directed perfusion
management?. Crit Care.

[10] Vercaemst L. (2008). Hemolysis in cardiac surgery patients undergoing cardiopulmonary
bypass: a review in search of a treatment algorithm. J Extra Corpor Technol.

[11] Spina S, Lei C, Pinciroli R, Berra L. (2019). Hemolysis and kidney injury in cardiac surgery: the protective
role of nitric oxide therapy. Semin Nephrol.

[12] Condello I. (2022). Magnetic levitation pumps for cell-free hemoglobin prevention
during VV ECMO. Crit Care.

[13] Dufour N, Radjou A, Thuong M. (2020). Hemolysis and plasma free hemoglobin during extracorporeal
membrane oxygenation support: from clinical implications to laboratory
details. ASAIO J.

